# Treatment of Salivary Gland Diseases: Established Knowledge, Current Challenges and New Insights

**DOI:** 10.3390/jcm11030802

**Published:** 2022-02-02

**Authors:** Konstantinos Mantsopoulos

**Affiliations:** Department of Otolaryngology, Head and Neck Surgery, Friedrich-Alexander-Universität Erlangen-Nürnberg (FAU), 91054 Erlangen, Germany; konstantinos.mantsopoulos@uk-erlangen.de; Tel.: +49-(0)9131-8533156; Fax: +49-(0)9131-8533833

In the last two decades, a change in paradigm has taken place in the management of salivary gland diseases. Hardly any type of pathological entity in this anatomical area has remained unaffected by the philosophy of individualised medicine, the general need for reducing therapeutic “invasiveness” and the necessity of achieving a satisfactory postinterventional quality of life. 

The need for reducing surgical invasiveness and achieving a better quality of life for patients has led to using minimal invasive surgical modalities in benign parotid gland lesions, focussing away from the dissection of the facial nerve and concentrating on the capsular features of the tumour itself. With respect to pleomorphic adenomas of the parotid gland, understanding the histological characteristics of the tumour capsule and the potential clinical relevance of their biological behaviour is fundamental for the management of these entities. Undoubtedly, the different histological subtypes, the various degrees of capsular intactness and the formation of pseudopodia, as well as satellite nodules, constitute a demanding profile with apparent clinical-surgical implications [1]. Historically, the surgical management of pleomorphic adenomas has followed a sinusoidal course from “enucleation” (dissection along the capsule or even opening the capsule and removing tumour material) in the 1940s (with recurrence rates of up to 45%) to standardised facial nerve dissection after the 1950s (with a high risk of iatrogenic injury of the facial nerve), resulting in a minimum of extracapsular dissection by the end of the last century. Remarkably, the surgical management of the same tumour in the parapharyngeal space tended to be consistent over the years, with the most common surgical modality in the management of parapharyngeal pleomorphic adenomas traditionally consisting of a transcervical, rather blunt dissection along the capsule of the tumour (e.g., with a peanut-swab holding forceps or even the surgeon’s finger), which is equivalent to the so-called enucleation of the same lesion in the parotid gland (that is not acceptable today). It seems that two completely different philosophies are not associated with a significant difference in the recurrence rate of the same lesion! The realisation of this apparent discrepancy gives rise to several questions (the histological similarity of these lesions in different anatomical localisations, potential overestimation of the role of the capsule) and sheds doubt on our state of knowledge about the actual mechanism of pleomorphic adenoma recurrence.

A thorough search of the salivary-gland-relevant literature reveals that research concerning the refinement of the surgical management of parotid tumours is undoubtedly advancing further. In this context, several reports refer to the value of facial nerve monitoring in both the intraoperative and the postoperative setting of parotidectomy. In everyday surgical routine, it is of major importance to investigate the correlation between a decrease in the electromyographic signal of the facial nerve and postoperative facial nerve function [2]. Interestingly, the expert’s opinion calls for facial nerve electrodiagnostics to become indispensable diagnostic tools in the detailed assessment of preoperative facial function in patients with head and neck neoplasms. These diagnostic modalities have been traditionally considered a means for detecting a subtle functional loss of the facial nerve, as well as indirectly identifying tumour dignity [3]. According to general surgical experience, it seems that the electroneurographic involvement of a clinically normal facial nerve due to a parotid gland tumour does not seem to be proof of nerve infiltration [3]. The need to further explore the status and significance of facial nerve electrodiagnostics in the preoperative phase should motivate further clinical research.

The paradigm shift in the treatment of salivary gland pathologies does not only refer to the management of parotid tumours. Expanding the use of sonography with a variety of ultrasound-assisted techniques and also new sialendoscopic-controlled modalities have led to the development of a significant number of treatment forms in obstructive sialopathy, aiming at preserving the anatomy and function of major salivary glands and reducing postinterventional morbidity [4]. It is impressive that the development of new minimally invasive and gland-preserving treatment modalities in cases with obstructive sialopathy have led to a reduction in the gland resection rate from 40–50% to less than 5%. New developments in the last decade, such as intraductal shock-wave lithotripsy, as well as the combination (e.g., sialendoscopy-assisted transoral duct surgery) and refinement of established techniques (retropapillary approach for distal parotid sialolithiasis), have partly replaced older, more invasive techniques (extracorporeal shock-wave lithotripsy, the combined transcutaneous–sialendoscopic approach) [5]. Undoubtedly, the use of the term “state-of-the-art” should be used very carefully when discussing a topic with such a potential for new developments and new techniques in the years to come!

While surgery remains the first-line treatment for salivary gland cancer, a multitude of systemic therapies including chemotherapy, targeted therapy and immunotherapy are available for inoperable and distant metastatic disease. However, there are a significant number of different studies dealing with the most common histological subtypes of salivary gland cancer that have not yet been reviewed and evaluated. A thorough search of the relevant literature reveals that systemic treatment can achieve prolonged progression-free and overall survival with a satisfactory quality of life, while the overall prognosis remains rather poor. In the future, further studies with a larger patient cohort and ideally only one histological subtype are needed in order to improve the outcome for salivary gland cancer patients. 

Given that clinical research on salivary glands is undoubtedly in progress and definitively advancing, the present Special Issue entitled “Treatment of salivary gland diseases: established knowledge, current challenges and new insights” is intended to provide an overview of recent advances in the management of salivary gland diseases in an effort to provide new evidence regarding several surgical and non-surgical aspects of this topic.
